# Annotations

**Published:** 1900-05-05

**Authors:** 


### ANNOTATIONS.

### Vinegar and Vitriol.

We are always pleased to find sanitary authorities bestirring themselves and putting in force their powers to prevent the sale of adulterated articles of diet. Still, even in such good deeds a little discretion is very useful, and we cannot but feel that this virtue was rather lacking in the proceedings recently taken by the vestry of St. James's, Westminster, in regard to the sale of certain samples of vinegar to which, it was alleged, sulphuric acid had been added beyond the amount permitted by law. It turned out, however, according to the Somerset House analysis, that the sulphuric acid which the vinegar contained was "chemically combined," in other words, the sulphuric acid existed in the form of sulphate of lime! Mr. Fenwick, in deciding the case, said that the prosecution urged that sulphuric acid had been added to the vinegar, inasmuch as the defendant had added sulphate of lime, and on the analysis the presence of sulphuric acid was disclosed in combination with lime. But he drew a very accurate and proper distinction between combined and uncombined sulphuric acid, and we cannot but think that the institution of a prosecution in such a case showed some want of discretion on the part of the vestry. If the addition of sulphuric acid to vinegar in the form of gypsum is the worst example of adulteration that the St. James's authorities can lay their hands upon, the inhabitants of that parish must be in a happy case in all that regards its food supply. But we don't believe it.

### The Growth of Boys.

Every year the general "weediness" which affects growing boys at certain stages of their development drives hundreds of anxious mothers to invent excuses for getting in a visit from the doctor that they may from his lips be reassured as to their darlings' condition. And it must be confessed that this is not always a matter on which it is easy to speak as positively as one would like to do. To examine the youngster thoroughly, and to make sure that so far as objective symptoms show there is no disease is comparatively simple. But to say whether he is "getting on" as he should do for his age is a much more difficult affair. To assist in the solving of this problem many tables of height, weight, and chest measurement are available. But, in truth, accurate as they may be, and full of information concerning the average boy, they tell nothing as to the individual boy before us, and it is the individual boy for whom alone the mother cares. In a paper[1] recently read before the Medical Officers of Schools Association, Mr. Cecil Hawkins, of Haileybury College, showed well the inutility of attempting to judge the individual by the average, and the incorrectness of assuming, as is too often done, that any marked departure from the average rate of growth or the average ratio of height, weight, and chest measurement is an unhealthy sign. Such a method does not take into consideration what he calls the natural scheme of the individual's growth. Of course, the difficulty is to find out in regard to the individual what this natural scheme is, and this Mr. Hawkins endeavours to overcome in a highly ingenious manner, which, if his tables should be confirmed by more extended observations, is likely to give much assistance in deciding the question. By dealing with larger numbers and arranging the boys at the several ages in what Mr. Francis Galton describes as percentile grades it is possible to map out with some approach to accuracy the rate of growth which may reasonably be expected from a boy who at any given age falls into any one of these grades or standards. When once the boy's grade is ascertained, by reference to the tables which Mr. Hawkins has constructed, one knows what may be expected of him. He ought to keep his grade. It is no use making oneself unhappy because a thin and weedy youth is not fat and plump like some of his companions. He may be far below the average in every particular yet still be keeping his health, and if year by year he steps into a higher percentile grade he is doing well. By frequent measurement, however, it ought to be possible to determine the general scheme of a boy's growth, and having "graded" him to discover very easily any departure from it. Such a record should of course be commenced young, and should be continued by the house master or the medical officer in the school to which the boy is sent.

[1] Published by Messrs. J. and A. Churchill.

### The Question of Teeth.

Much disappointment has been felt by many candidates for army employment at being rejected on medical examination, and among the various reasons assigned for such rejection none has perhaps caused more astonishment and disgust among otherwise healthy candidates than the loss of teeth. But the rule that no one is to be accepted who has lost ten teeth is surely a proper one, especially when it is remembered that, according to the official "requirements" as regards the teeth of candidates for commissions, decayed teeth if well filled are considered as sound. People, no doubt, get on very well at home with a small number of teeth, and many struggle on without experiencing much detriment even when such teeth as they possess are far from perfect. But it is another matter in the rough and tumble of war. The question does not hinge, as some have supposed, on the loss of teeth being an indication of constitutional weakness; for, as is well known, it is just as likely to arise from imperfect dentistry and a nature impatient of pain. The real point is as to the power of a comparatively toothless man to bear the hardships of war; and, in regard to this, there can be no doubt that he stands at a disadvantage. Put it as one will, the loss of ten teeth must seriously interfere with the power of mastication, and must, to the same degree, render a man dependent on his cook—a very serious drawback where for weeks together only the roughest food may be available. Again, although it is maintained by dentists that with well-fitting artificial teeth masticatory power is perfectly restored, one has to remember that people with such appliances, as well as those who possess carious teeth, are far more dependent upon the tooth-brush than is the case with those whose teeth are as Nature made them. A time may come, perhaps, when it may be necessary to accept candidates whose efficiency in the field depends upon spectacles, false teeth, tooth brushes, and dinner pills. But that time is not yet.

